# New Approaches to the Role of Thrombin in Acute Coronary Syndromes: *Quo Vadis* Bivalirudin, a Direct Thrombin Inhibitor?

**DOI:** 10.3390/molecules21030284

**Published:** 2016-02-27

**Authors:** María Asunción Esteve-Pastor, Diana Hernández-Romero, Mariano Valdés, Francisco Marín

**Affiliations:** Department of Cardiology, Hospital Universitario Virgen de la Arrixaca, Instituto Murciano de Investigación Biosanitaria, IMIB-Arrixaca, Murcia 30120, Spain; masunep@gmail.com (M.A.E.-P.); dianahr@um.es (D.H.-R.); mvch@valdeschavarri.e.telefonica.net (M.V.)

**Keywords:** bivalirudin, STEMI, non-STEMI, ACS, thrombin

## Abstract

The pathophysiology of acute coronary syndrome (ACS) involves platelet activation and thrombus formation after the rupture of atherosclerotic plaques. Thrombin is generated at the blood-plaque interface in association with cellular membranes on cells and platelets. Thrombin also amplifies the response to the tissue injury, coagulation and platelet response, so the treatment of ACS is based on the combined use of both antiplatelet (such as aspirin, clopidogrel, prasugrel and ticagrelor) and antithrombotic drugs (unfractionated heparin, enoxaparin, fondaparinux and bivalirudin). Bivalirudin competitively inhibits thrombin with high affinity, a predictable response from its linear pharmacokinetics and short action. However, a present remarkable controversy exists between the latest main Guidelines in Clinical Practice and the key trials evaluating the use of bivalirudin in ACS. The aim of this review is to update the development of bivalirudin, including pharmacological properties, obtained information from clinical trials evaluating efficacy and safety of bivalirudin in ACS; as well as the recommendations of clinical Guidelines.

## 1. Introduction

Acute coronary syndromes (ACS) are the leading cause of death and high morbidity worldwide despite advances in pharmacological and interventional treatment. The genesis of ACS disrupts molecular mechanisms of hemostasis involving platelet activation, aggregation and thrombin generation as the final phase after activation of coagulation system [1]. Thus, the combination of antiplatelet (aspirin, clopidogrel, prasugrel and ticagrelor) and anticoagulant (unfractionated heparin (UFH), fondaparinux, enoxaparin and bivalirudin) agents is essential in ACS treatment [2]. Despite the administration of optimal antiplatelet therapy, 9%–11% patients with ACS remain at risk of recurrent ischemic cardiovascular events as a result of thrombin generation [3]. Medical strategies have been focused on thrombin inhibition, since this molecule is, as explained, a central actor in arterial thrombogenesis [4,5,6]. UFH has been widely considered as the anticoagulant of election. However, due to its limitations in arterial thrombosis, a broad spectrum of different anticoagulants has been arisen. New studies have been performed suggesting that inhibition of factor Xa offers more advantages over targeting thrombin, because one molecule of factor Xa has the potential to generate 1000 molecules of thrombin [7,8]. Numerous clinical trials have been conducted to identify the optimum pharmacological treatment to avoid ischemic and bleeding events in patients with ACS undergoing percutaneous coronary intervention (PCI), including oral and parenteral agents [9]. The main target is to find an effective anticoagulant therapy without severely increasing rates of bleeding [10].

Bivalirudin is a direct thrombin inhibitor that has a near immediate effect with linear dose response, resulting in a predictable anticoagulant effect. In several studies bivalirudin reduced ischemic and bleeding events compared with heparin [11], but the latest trials and clinical guidelines, the role of this parenteral agent in ACS, especially in ST elevation myocardial infarction (STEMI) patients is controversial due to high rate of early stent thrombosis [12]. On the other hand, the use of direct oral anticoagulants (DOACs) such as dabigatran, apixaban, rivaroxaban or edoxaban is attractive but they are not yet ready to be used in ACS [13]. In this review, we analyze the role of thrombin in the ACS, the molecular properties of bivalirudin, the main clinical trials and the controversy arisen with current clinical guidelines.

## 2. Pathophysiology of ACS and the Role of Thrombin

Initiation of ACS is caused by disruption of atherosclerotic plaques and exposition of high thrombogenic matrix components that contact with platelets. These platelets present constitutive receptors (GPVI and integrin α2β1) that adhere to subendothelial collagen and von Willebrand factor (vWF) [14]. Activation of adherent platelets promotes release of thromboxane A2 and adenosine diphosphate, that in turn may activate neighboring platelets via sensitive receptors P2Y, and TPa located on the platelet surface. In addition, platelet activation induces conformational changes in the surface integrin glycoprotein IIb/IIIa, leading to fibrinogen and vWF binding that bridge the platelets together resulting in platelet aggregation [14]. Aggregated platelets decrease blood flow and increase local shear forces.

**Figure 1:**
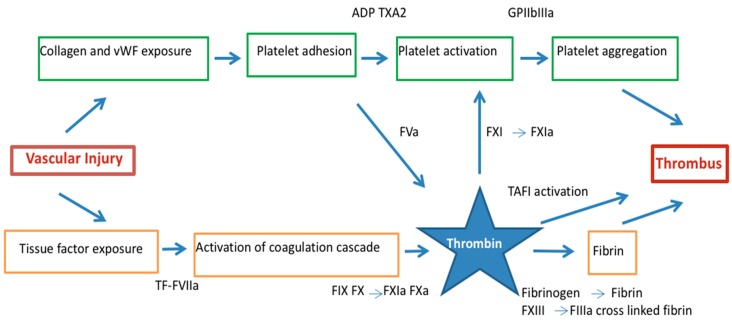
Thrombus formation in acute coronary syndrome by the dual pathway: platelet activation/aggregation and fibrin formation. Thrombin links vascular injury, platelet activation and the conversion of fibrinogen to fibrin. ADP = adenosine diphosphate, F = factor, GP = glycoprotein, α2β = integrin α2β, TF = tissue factor, TXA2 = thromboxane A2, PAR = protease activated receptor, vWF = von Willebrand factor.

Upon activation, platelets increase expression of anionic phospholipids on their surface and promote coagulation cascade by assembling of coagulation factor complexes. Concomitant with platelet activation and aggregation, plaque disruption exposes tissue factor (TF) expressed by lipid-laden macrophages and smooth muscle cells found in the core of the disrupted plaque that results in activation of the coagulation system [15,16,17] (Figure 1).

Exposed tissue factor binds activator FVII and this complex activates FIX and X in the presence of calcium and their cofactors on the platelets surface FVIIIa and Va, respectively to form tenase and prothrombinase [18,19]. Hence, platelets have an important role in amplifying thrombin generation that induces thrombus formation. Thrombin is the principal activator of platelets at sites of injury activating effects via protease-activated receptors (PARs), most importantly PAR-1 in human platelets [20,21,22] (Figure 1).

Thrombin converts fibrinogen to fibrin serving as matrix scaffold at the vascular injury that lead to the formation of platelet-rich thrombi, the central event in the pathogenesis of ACS [23,24]. During this process, thrombin remains enzymatically active and binds to fibrin where is protected from inactivation by fluid-phase inhibitors [25]. In addition, thrombin is a potent platelet agonist by amplifying its generation via the activation of FV, FVIII and FXI [14,26] further enhancing coagulation amplification and propagation [27]. Thrombin also activates FXIII that performs fibrin cross-linking rendering the thrombus structure more resistant to degradation [28]. The cross-linking of α2-antiplasmin, the major plasmin inhibitor, onto fibrin, and the activation of a latent carboxypeptidase B, known as thrombin activatable fibrinolysis inhibitor (TAFI) [29] also contributes to this stabilization, since this enzyme attenuates fibrinolysis, presumably by removing carboxy-terminal lysine residues from fibrin, thereby preventing the binding of plasminogen and plasmin [30]. Moreover, lysis of coronary thrombus also induces thrombin formation. Clotting is promoted when plasmin is generated by thrombolysis, that activates contact factors [31] and convert prothrombin to thrombin [32,33].

Hence, thrombus formation in the context of the ACS is coordinated by two different pathways led by platelet and thrombin [28]. Under high shear conditions, formed thrombi appear platelet-rich and, in contrast, under low blood flow conditions, fibrin-rich thrombi are generated [34]. Both pathways are interconnected at different levels and conditioned by environmental conditions with the result of a mix thrombus with a platelet-rich head and fibrin-rich tail in coronary arteries [28].

Thrombin presents different specific binding sites for several substrates. It contains an active catalytic site with serine residues responsible for the proteolytic cleavage of clotting factors that become activated. In addition thrombin contains a substrate recognition site, the exosite 1, which orients fibrinogen to bind thrombin to fibrin in a clot. Also, a second positively charged region, exosite 2, is the heparin-binding site [35].

Indirect thrombin inhibitors, direct thrombin inhibitors and PAR-1 antagonists in addition to standard antiplatelet therapy have been evaluated in ACS treatment. Vorapaxar, an oral PAR-1 antagonist that inhibits thrombin-induced platelet activation, was recently investigated in patients with ACS. In their main clinical trial, TRACER trial [36], there was no reduction in ischemic events (18.5% *vs.* 19.9%. HR 0.92, 95% CI 0.85–1.01; *p =* 0.07) but bleeding rates were increased (7.2% *vs.* 5.2%. HR 1.35, 95% CI 1.16–1.58; *p <* 0.001).

Indirect thrombin inhibitors such as UFH and low molecular weight heparin (LMWH) act by enhancing the function of antithrombin III (ATIII), which indirectly leads to inactivation of thrombin and factor Xa [37]. UFH has been the anticoagulant treatment of choice during decades for blocking thrombin generation and activity; it presents several limitations that are overcome by direct thrombin inhibitors. UFH consists of a family of highly sulfated polysaccharide chains, ranging in molecular weight from 3000 to 30,000 Dalton (Da). Each molecule of UFH has a binding site for factor Xa, thrombin or both and the antithrombin activity of UFH depends on the activation of antithrombin, with inactivates thrombin [38]. Moreover, UFH is easily administered, has rapid onset, easily measurable efficacy (monitored by activated clotting time (ACT)) and reversibility with protamine sulphate, but treatments with UFH produce complications like osteoporosis and heparin-induced thrombocytopenia or platelet activation, with a dangerous prothrombotic condition [39].

Because of the limitations of UFH, LMWH have emerged [39]. LMWH (*i.e*., enoxaparin) treatment produces more uniform and consistent anticoagulation than UFH because it is less plasma protein binding leading to more predictable bioavailability. LMWH has more favorable pharmacokinetics and produces greater proximal inhibition of the coagulation cascade, in particular factor XA, without continuous monitoring [40]. Several trials (ExTract-TIMI 25 [41] or the STEEPLE trial [42]) showed that enoxaparin is superior to UFH in reducing ischemic events with a better safety profile.

Fondaparinux is a synthetic pentasaccharide that binds antithrombin, resulting in a 340-fold increase in the rate of FXa inhibition over the basal rate [13]. Two large clinical trials have been published in which fondaparinux was evaluated in ACS. OASIS-5 trial [43] evaluated the role of fondaparinux in comparison with enoxaparin in non-STEMI patients The authors concluded that fondaparinux was similar to enoxaparin in reducing the risk of ischemic events (5.8% *vs.* 5.7%) with a reduction of major bleeding events (2.2% *vs.* 4.1%; *p <* 0.001). The OASIS-6 trial [44] evaluated the role of fondaparinux in comparison with enoxaparin in STEMI patients. In patients that underwent percutaneous coronary intervention (PCI), the rate of death and myocardial infarction did not differ significantly between the two groups (93 *vs.* 114) and rates of severe bleeds were similar (9 *vs.* 16) but there was higher rate of guiding catheter thrombosis (0 *vs.* 22; *p <* 0.001). Due these clinical trials, fondaparinux is recommended as anticoagulation treatment for non-STEMI patients but not for STEMI patients undergoing PCI.

Direct anticoagulants produce inhibition of both fibrin-bound thrombin and fluid-phased thrombin with a predictable anticoagulant response [45]. Direct anticoagulants including direct Xa inhibitors (apixaban, rivaroxaban and edoxaban) and direct thrombin inhibitors (hirudin, bivalirudin and dabigatran).

In the last decade, four direct oral anticoagulants (DOACs) have emerged. These are small molecules that bind directly to their intended target, either activated factor IIa-thrombin (dabigatran) or activated factor Xa (rivaroxaban, apixaban or edoxaban), and neutralize their activity [46]. Their pharmacology is well characterized. All of them are rapidly absorbed following oral administration and have a relatively short half-life (5–17 h). The differential pharmacokinetic characteristics of dabigatran in comparison with direct FXa inhibitors are its low bioavailability (6%) despite the prodrug strategy followed to enhance its intestinal absorption, its high renal clearance (85% of absorbed dose), and its low metabolism. Direct FXa inhibitors have a good oral bioavailability (>50%), a lower renal clearance than dabigatran, although still significant (54%–73% of absorbed dose is excreted through the kidneys) and undergo extensive metabolism by mainly CYP3A4 (rivaroxaban), CYP3A4/5 (apixaban) and/or hydrolysis (edoxaban) [47].

In atrial fibrillation (AF), DOACs have emerged as an alternative for vitamin K antagonists (VKAs) for thromboembolism prevention and they are at least as effective as VKAs in prevention ischemic stroke and also associated with fewer intracranial hemorrhages and less drug interactions [48,49].

A recent meta-analysis evaluated the role of DOACs and PAR-1 antagonists in the treatment of ACS. The study revealed an up 3-fold increased risk of hemorrhagic stroke in patients receiving DOACs (OR 3.45, 95% CI 1.62–7.37, *p =* 0.001, I^2^ = 0%) or PAR-1 antagonists (OR 2.60, 95% CI 1.18–5.69, *p =* 0.02, I^2^ = 0%) in addition to antiplatelet therapy with only moderate reduction of composite of death, myocardial infarction or stroke [3]. Thereby, although DOACs had an up to 50% reduction in intracranial hemorrhage compared to VKAs in AF patients, this meta-analysis showed that DOACs added to antiplatelet therapy still let to a markedly increased risk of hemorrhagic stroke in patients with ACS.

For parenteral anticoagulation treatment, hirudin, a 65 amino-acid polypeptide, is the prototype of direct thrombin inhibitors, first isolated from the salivary glands of the leech *Hirudo medicinalis* [50]. The part of the structure containing the domains needed for thrombin binding and inhibition were studied for developing smaller synthetic molecules as bivalirudin [51].

## 3. Bivalirudin Mechanism of Action

**Figure 2:**
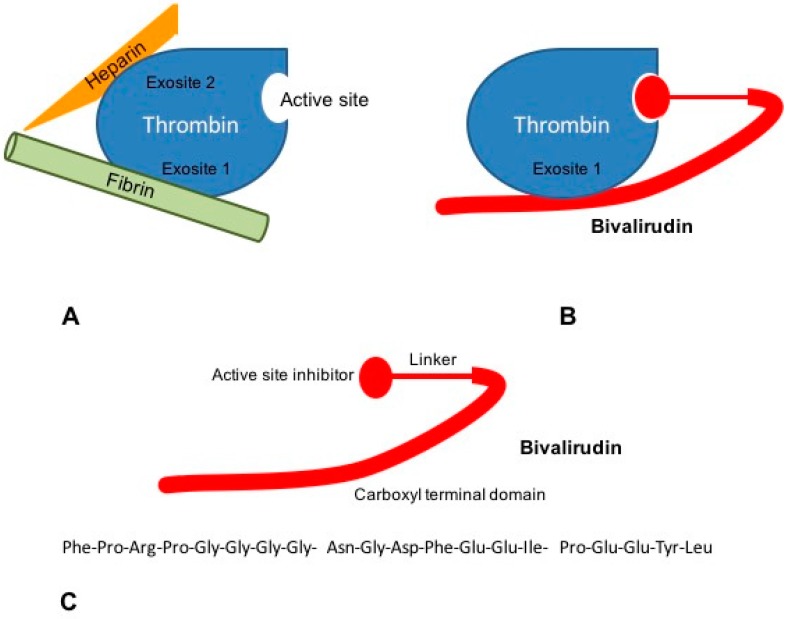
Mechanisms of action of bivalirudin. (**A**) The ternary heparin-thrombin-fibrin complex increases the affinity of thrombin for its fibrin substrate. Heparin binds thrombin via exosite 2, while interaction of thrombin to fibrinogen occurs through exosite 1; (**B**) Bivalirudin binds to thrombin both via the active site and exosite 1, blocking the interaction between thrombin and fibrinogen; (**C**) Bivalirudin consists of 20 aminoacids disposed in three structural domains: a catalytic site domain, a carboxyl terminal region and a linking domain.

Bivalirudin is a 20-aminoacid parenteral synthetic analog of hirudin that interacts directly with both the active site and the substrate binding of thrombin [52]. Its structure is divided in a C-terminal domain linked by four glycine residues to a N-terminal domain. The carboxyl terminal region contains 12-aminoacids with the binding region to exosite 1 of thrombin. Four glycine residues bridge C-terminal and N-terminal domains. The amino-terminal region is composed of a 4-aminoacid sequence (D-Phe-Pro-Arg-Pro) which has been reported to bind the catalytic site of thrombin [51] (Figure 2).

Bivalirudin binds thrombin by the active site and exosite 1 whereas heparin interacts with thrombin via the exosite 2 (Figure 2). Bivalirudin-thrombin complex interaction starts as a non-competitive inhibition. Once combined with thrombin, the drug is slowly cleaved, by the Arg3-Pro4 bond in the amino-terminal region, hence allowing the enzyme to restore its haemostatic function [53,54]. The carboxyl-terminal region of bivalirudin remains bound to exosite 1 with a lower affinity. At this moment, the interaction changes into a competitive binding, producing a transient inhibition of thrombin [55].

## 4. Bivalirudin Pharmacokinetics and Pharmacodynamics

Bivalirudin does not bind to plasma proteins or to red blood cells [38,56]. It is cleared with a mean total body clearance of 3.4 mL/kg per minute and the elimination half-life is over 25 min in patients with normal renal function (glomerular filtration rate, GFR ≥ 60 mL/min). Bivalirudin clearance decreases in patients with impaired renal function, being 34 and 57 min in patients with moderate (GFR 30–59 mL/min) or severe renal function (GFR 10–29 mL/min), respectively [57,58].

The pharmacodynamics of bivalirudin are determined by its anticoagulant effect assayed by prothrombin time, activated clotting time (ACT) or activated partial thromboplastin time (APTT) [57]. Several studies and clinical trials have been performed for establishing the relationship between the anticoagulant response and bivalirudin dose.

Robson *et al.* reported results in 25 patients undergoing PCI divided in three groups of different renal function (normal, mild and moderate renal impairment). Patients received a bolus dose of bivalirudin (1 mg/kg) followed by an infusion (2.5 mg/kg per hour for 4 of 6 h, then 0.5 mg/kg per hour for 4 of 6 h). They also performed an independent study in eight patients with severe renal impairment receiving a bivalirudin bolus of 1 mg/kg, followed by an infusion of 0.5 mg/kg per hour for 10 h. The authors analyzed bivalirudin in plasma and urine by highly specific liquid chromatography-mass spectrometry assay. They concluded that the pharmacokinetics of bivalirudin are dependent on renal function but independent of dose and gender. Approximately 20% of unchanged drug was cleared via the kidney, and the remainder presumably underwent proteolysis intracellularly. The pharmacodynamics of bivalirudin resulted dose-dependent and gender-independent. They found bivalirudin kinetics linear in the dose ranges used in PCI, which are nowadays under controversy for use in ACS [59].

A dose-ranging study performed in 55 patients with unstable angina evaluated bivalirudin dosage relationship with the prolongation of the APTT and a close correlation was reported for doses ranging from 0.25 to 1.0 mg/kg per hour. Intra- and interindividual variation coefficient ranged from 6.2%–11.9% and 11%–20%, respectively [60]. Topol *et al.* reported a similar dose-response ratio in APTT and ACT evaluated in different groups of patients receiving between 0.6 to 2.5 mg/kg bivalirudin per hour in a clinical study including 291 patients undergoing coronary angioplasty [61]. Another study carried out by Lui *et al.* evaluated ACT levels in 12 patients receiving 1.0 mg/kg bivalirudin per hour, followed by an infusion of 2.5 mg/kg/per hour. They reported an average ACT of 362 s at steady state and a maximal ACT levels > 300 s in all patients [62]. The same bivalirudin administration was used in the Hirulog Angioplasty Study (HAS) carried out in 4312 patients undergoing angioplasty for unstable or postinfarction angina. The study reported a median ACT of 346 s [63].

Since a poor relationship has been reported for the activated clotting time (ATC) or activated partial thromboplastin time (APTT) with the concentration of r-hirudin as measured by a chromogenic factor II assay, an alternative method has been proposed for hirudin derivatives. Bivalirudin can be monitored at the point of care via ecarin clotting time (ECT), a whole blood clotting assay based on the prothrombin–activated snake venom ecarin, which offers reliable on-line monitoring for direct thrombin inhibitors [64]. This method has been investigated for the use of bivalirudin during cardiopulmonary bypass (CPB), suggesting that ECT may be a useful assay for monitoring bivalirudin during CPB, enabling precise control of the drug [65].

Another study assessed pharmacokinetics and pharmacodynamics following intravenous administration of bivalirudin in healthy Chinese subjects equally divided into four groups of different bivalirudin dosage. The study was a randomized, single-blind and placebo-controlled (bivalirudin groups: *n* = 9/group; placebo groups: *n* = 3/group) design. They determined a half-life of bivalirudin of 34 min, similar to the reported data of Caucasian patients and it can provide the desired anticoagulant effects. They reported a strong correlation between bivalirudin concentration and anticoagulant effect and established a sigmoid model to fit the pharmacodynamic parameters activated clotting time (ACT), activated partial thromboplastin time (APTT) and prothrombin time (PT) and bivalirudin concentrations. The findings of this study suggested that the same dosing regimens of bivalirudin may be administered to Chinese and Caucasian patients [66].

A randomized controlled clinical trial in healthy volunteers showed a significant correlation between pharmacokinetic and pharmacodynamic variables for both intravenous (*r* = 0.8, *p* < 0.001) and subcutaneous administration (*r* = 0.7, *p* = 0.002). The intravenous infusion of hirulog showed a rapid, dose-dependent prolongation of APTT, PT, and thrombin time (TT). Plasma levels are reached within 2 min after intravenous administration and there was a corresponding dose-dependent increase in plasma hirulog levels, with absolute bioavailability between 40% and 80% [67].

## 5. Bivalirudin in Acute Coronary Syndromes

Bivalirudin has been studied in several randomized clinical trials in STEMI and non-STEMI patients undergoing PCI with contradictory findings (Table 1 and Table 2). For STEMI patients, five clinical trials have evaluated the role of bivalirudin in primary PCI. Harmonizing Outcomes with Revascularization and Stents in Acute Myocardial Infarction (HORIZONS-AMI) was the first clinical trial to evaluate bivalirudin *vs.* heparin treatment in STEMI patients [68]. This open label study compared bivalirudin alone with UFH plus glycoprotein IIbIIIa inhibitors (GPIs) and showed that patients in the bivalirudin group had a significantly reduced rate at 30 days of net adverse clinical events (combination of major bleeding or major adverse event (death, reinfarction, target vessel revascularization for ischemia or stroke) with 9.2% *vs.* 12.1% for UFH group (RR 0.76, 95% CI 0.63–0.92; *p* = 0.005). Also, bivalirudin achieved a lower rate of major bleedings with 4.9% *vs.* 8.3% (RR 0.60, 95% CI 0.46–0.77; *p* < 0.001). Therefore, treatment with bivalirudin as compared with UFH improved event free survival at 30 days with lower major bleeding. However, bivalirudin infusion was stopped just when coronary intervention finished, featuring a higher rate of early stent thrombosis in patients treated with bivalirudin (1.3% *vs.* 0.3%; *p* < 0.001).

Similar findings were observed in the European Ambulance Acute Syndrome Angiography (EUROMAX) randomized trial. In this study, patients with STEMI were randomized to initiate bivalirudin or UFH with optional GPIs during transport in ambulance for primary PCI [69]. All patients underwent angiography within the first two hours after first medical contact. The primary endpoint at 30 days (composite of death and major bleeding no associated with CABG) occurred in 5.1% with bivalirudin and 8.5% in UFH group (RR 0.60, 95% CI 0.43–0.82; *p =* 0.001) and bivalirudin reduced the risk of major bleeding (2.6% *vs.* 6.0% respectively; RR 0.43, 95% CI 0.28–0.66; *p <* 0.001). Regardless in EUROMAX trial the use of radial access site and new P2Y_12_ inhibitors such as ticagrelor or prasugrel was higher than previous trials, stent thrombosis was more frequent in the bivalirudin group than in control group (1.6% *vs.* 0.5% RR 2.89, 95% CI 1.14–7.29; *p* = 0.02). Although 93% of patients in bivalirudin group received a prolonged (4 h or longer) infusion of bivalirudin compared with HORIZONS-AMI trial [68] where the infusion was only during PCI, stent thrombosis occurred within first 24 h (*p* = 0.007) with no significant difference in rates of subacute stent thrombosis [70].

Inside this concern for early stent thrombosis, divergent findings emerged from How Effective Are Antithrombotic Therapies in Primary Percutaneous Coronary Intervention (HEAT-PPCI) single-center trial [71]. The authors compared bivalirudin with UFH, and similar proportion of patients in both groups received GPIs. The primary efficacy outcome (composite of all-cause mortality, cerebrovascular accident, reinfarction and unplanned target lesion revascularization) was reported as 8.7% in bivalirudin group and 5.7% in heparin group (RR 1.52, 95% CI 1.09–2.13; *p* = 0.01). No differences were observed in the major bleeding rate, comparing bivalirudin vs UHF groups (3.5% *vs.* 3.1% respectively, RR 1.15, 95% CI 0.70–1.89; *p* = 0.59). Furthermore, the rate of early thrombosis stent was higher from bivalirudin group than for UFH group (3.4% *vs.* 0.9% respectively; RR 3.91; 95% CI 1.61–9.52; *p* = 0.001). The HEAT-PPCI trial concluded that UFH reduces the incidence of major adverse ischemic events in patients with STEMI underwent primary PCI, with lower rate of stent thrombosis and similar rates of major bleeding events compared with bivalirudin. As a result, there was a significant change in clinical guidelines for STEMI management about treatment with bivalirudin and generated mistrust between professionals regarding the use of bivalirudin.

Then, to determinate the safety and efficacy of bivalirudin, the Bivalirudin in Acute Myocardial Infarction Versus Glycoprotein IIb/IIIa and Heparin Undergoing Angioplasty (BRIGHT) trial was conducted [72]. Patients were randomized to bivalirudin treatment, UFH alone or UFH plus GPIs treatment. Also, after PCI procedure finished 1.75 mg/kg/h infusion of bivalirudin was administered for median of 180 minutes and a reduced dose infusion of bivalirudin (0.2 mg/kg/h) for up 20 h could be administered at physician discretion. Approximately 78% of primary PCI were performed transradially with 99% of employment of drug-eluting stent. Net adverse clinical events incidence (NACE) at 30 days was lower in bivalirudin group compared with UFH (8.8% *vs.* 13.2%; RR 0.67; CI 95% 0.50–0.90; *p* = 0.008). The 30-day bleeding rate was 4.1% for bivalirudin, 7.5% for UFH and 12.3% for UFH plus GPIs (*p* < 0.001) Furthermore, this is the only one of last trials that rates of acute stent thrombosis were not increased with bivalirudin (0.6% *vs.* 0.9% *vs.* 0.7%, respectively, *p* = 0.77). Another small trial, the Bavarian Reperfusion Alternatives Evaluation (BRAVE-4) trial analyzed the benefit of bivalirudin plus prasugrel *vs* UFH plus clopidogrel for STEMI patients [73]. The trial was unable to demonstrate significant differences in NACE between those groups because the study was stopped prematurely due to slow recruitment.

For non-STEMI patients, the clinical trial findings are more consistent. The first highlighted study was PROTECT-30 which evaluated the role of monotherapy with bivalirudin compared with UFH plus GPIs among patients with non-STEMI [74]. The authors concluded that the coronary flow reserve was significantly greater with bivalirudin group (1.43%) than UFH-GPIs group (1.33%) in moderate-high risk patients with non-STEMI (*p =* 0.036) and UFH-GPIs had higher minor bleeding (2.5% *vs.* 0.4%, *p =* 0.027) and transfusion (4.4% *vs.* 0.4%, *p <* 0.001) than the bivalirudin group.

Nevertheless, the main clinical trial in non-STEMI patients was the Acute Catheterization and Urgent Intervention Triage strategy (ACUITY) trial. Non-STEMI patients (13,819) were randomized 1:1:1 to receive bivalirudin alone, bivalirudin plus GPIs or UFH plus GPIs [75]. Of all enrolled patients, only 7789 (56%) were managed with PCI. Angiography was performed by protocol within 72 h after randomization with 6% of radial access, and prasugrel or ticagrelor were not available for use during this trial. All antithrombin treatments were discontinued at the end of PCI. There was no significant difference in NACE at 30 days (composite of ischemia (death from any cause, myocardial infarction or unplanned revascularization of ischemia) or major bleeding between bivalirudin group (15% *vs.* 13%, RR 1.12 CI 95% 0.98–1.28; *p* = 0.10) but the rates of major bleeding were significantly fewer in patients with bivalirudin alone (4%) among those who received UFH plus GPIs (7%) (RR 0.52, 95% CI 0.40–0.66; *p <* 0.0001). In addition, stent thrombosis within first 30 days occurred in 1.4% patients with similar rates among the three treatment groups (*p* = 0.87). Thus the authors concluded that in moderate and high-risk patients with non-STEMI, anticoagulation with bivalirudin alone during PCI showed similar rate of NACE and stent thrombosis than UFH treatment with significantly lower risk of major bleeding events. In the same way, the Intracoronary Stenting and Antithrombosis research (ISTAR-REACT 4) trial analyzed the role of bivalirudin in non-STEMI patients who had underwent PCI [76]. In this trial, 1721 patients were randomized 1:1 to receive UFH plus GPIs (abciximab) *vs.* bivalirudin alone immediately before PCI. All patients also received antiplatelet treatment with aspirin 325 to 500 mg and 600 mg of clopidogrel and underwent to invasive intervention within 24 h after admission. Importantly, radial artery access was used only in six patients. The primary endpoint at 30 days (a composite of death, large recurrent myocardial infarction, urgent target vessel revascularization or major bleeding) was 10.9% in UFH-GPIs group and 11.0% in bivalirudin group (RR 0.99, 95% CI 0.74-1.32; *p* = 0.94). Major bleeding was lower in bivalirudin group (2.6%) compared with 4.6% in UFH-GPIs treatment (RR 1.84, 95% CI 1.10-3.07; *p* = 0.02) and there were no differences between definite stent thrombosis (0.6% *vs.* 0.7%). Therefore, UFH plus GPIs failed to reduce the primary endpoint and increased the risk of bleeding events with similar rate of stent thrombosis in non-STEMI patients.

## 6. Clinical Guidelines Management of Antithrombotic Treatment in STEMI and Non-STEMI Patients

Due to the contradictory results reported in previous clinical trials in STEMI patients, there is a change in the recommendations about the use of bivalirudin between the current and previous clinical guidelines (Table 3). In 2012 European Society of Cardiology (ESC) STEMI guidelines [77], bivalirudin is recommended over UFH and GP IIb/IIIa blocker with high evidence (class I, Level B). From the American guidelines (AHA/ACC), bivalirudin treatment had similar evidence of recommendation [78]. NICE guidelines published in July 2013 recommended Bivalirudin in combination with aspirin and clopidogrel for the treatment of adults with STEMI undergoing primary PCI [79].

However, due to the findings of HEAT-PPCI trial [71] that UFH reduces the incidence of major adverse ischemic events with lower incidence of early stent thrombosis and no difference in major bleedings, the new 2014 ESC guidelines of myocardial revascularization changed their recommendation to evidence Class IIa, Level A in patients at high risk of bleeding [80]. The revascularization guidelines emphasize that recent clinical trials showed an excess risk of acute stent thrombosis with bivalirudin while differences in major bleeding are small.

As happened with clinical trials, the recommendations of management of non-STEMI patients are consistent about different clinical guidelines, with high level of evidence for use bivalirudin. NICE clinical guidelines [81] published in March 2010 recommended bivalirudin for non-STEMI patients undergoing PCI as an alternative treatment to the combination of UFH plus GPI, especially in patients at intermediate or higher risk of adverse cardiovascular events and which are not already receiving GPI or fondaparinux [80].

The new 2015 ESC guidelines for the management of non-STEMI recommend bivalirudin as an alternative to UFH plus GPIs inhibitors during PCI with class I-level A of evidence [82]. In 2014 American guidelines, bivalirudin is recommended until diagnostic angiography or PCI with class I-level B like 2014 ESC revascularization guidelines that recommend bivalirudin as alternative to UFH plus GPIs with class I-level A of evidence [80,83].

## 7. New Evidence–New Approaches

In the last year, there have been new interesting publications to help us in decision-making about treatment with bivalirudin. Valgimigli *et al.* conducted the Minimizing Adverse Haemorrhagic Events by TRansradial Access Site and Systemic Implementation of angioX (MATRIX) trial [84]. This study was the first powerful trial with 7213 patients and including current medical advances in STEMI patients. The authors showed that radial access reduces net adverse clinical events (8.8% *vs.* 10.3%, RR 0.67 95% IC 0.49–0.92; *p =* 0.013). Also they compared standard care of ESC guidelines with UFH plus GPIs with bivalirudin alone. Antiplatelet treatment included clopidogrel, prasugrel and ticagrelor. There were two bivalirudin groups: short bivalirudin arm, when the infusion stopped at the completion of PCI and long bivalirudin arm where the dose infusion reduced to 0.25 mg/kg/h for at least six hours. Bivalirudin was not statistically superior to UFH at 30 days for MACE (10.3% *vs.* 10.9%, RR 0.94, 95% CI 0.81–1.10, *p* = 0.45) or NACE (11.2% *vs.* 12.4%; RR 0.89, 95% CI 0.78–1.10, *p* = 0.12) but the superiority of bivalirudin was in reducing major bleeding. As previous trials in STEMI patients, early stent thrombosis was higher in bivalirudin group (1.0% *vs.* 0.6%, RR 1.71. 95% CI 1.00–2.93, *p =* 0.048). In a *post-hoc* analysis of the MATRIX trial, the authors found no evidence for an interaction between the effect of radial *vs.* femoral access and allocation to bivalirudin or UFH treatment group for all cause mortality, MACE or major bleeding (p for interaction ≥0.64). Currently, there are no other major clinical trials, so meta-analysis were performed with the mail clinical trials for STEMI patients (sample size between 1000 and 3600 patients). Farag. *et al.* [85] performed a meta-analysis with randomized clinical trials with bivalirudin *vs* UFH in ACS and concluded that bivalirudin increases early stent thrombosis in STEMI patients with small reduction difference in major bleeding with bivalirudin, and the reduction in major bleeding with bivalirudin is not related to vascular access site. In the same way, Bittl *et al.* [86] analyzed all clinical trials about bivalirudin in STEMI and non-STEMI clinical scenarios, and quantified the effect of vascular-access selection and newer potent oral P_2_Y_12_ blockers on the risk of bleeding and stent thrombosis during PCI. The weighted mortality rate was no different with 2.18% for bivalirudin and 2.34% with heparin (OR 0.93, 95% CI 0.77–1.14; *p =* 0.295). The absolute bleeding rate of 3.52% for bivalirudin was lower than the rate of patients treated with heparin (5.39%) while the absolute rate of stent thrombosis was higher with bivalirudin (1.40%) than with UFH (0.90%). In addition, the important findings of this meta-analysis was that no significant bleeding advantage of bivalirudin over UFH could be identified in randomized clinical trials with radial access (OR 0.80, 95% CI 0.57–1.41) and the use of prasugrel or ticagrelor eliminated bleeding differences of bivalirudin (OR 0.80; IC 95% 0.63–1.03) but did not reduce the risk of stent thrombosis in the bivalirudin arm (OR 2.20 IC 95% 1.48–3.27).

## 8. Questions to Be Answered

Old clinical trials related early stent thrombosis with the characteristics of the stent (bare metal *vs* drug-eluting), the lack of power P_2_Y_12_ antiplatelet treatment or absence of infusion of bivalirudin after PCI in the trials [87]. In recent clinical trials, despite prolonged bivalirudin infusion up to 20 h and the use of ticagrelor or prasugrel, bivalirudin shows higher rates of early stent thrombosis [88]. Moreover, the use of more potent and rapidly acting P_2_Y_12_ inhibitor reduces the bleeding advantage of bivalirudin over UFH, even when using radial access with lower rate of bleeding. Generally, STEMI patients undergo to PCI in the first 2–4 h, with simultaneous administration of antiplatelet and antithrombotic treatment. The more potent P_2_Y_12_, prasugrel and ticagrelor need at least 4–6 h after administration to achieve maximum effect. The discontinuation of bivalirudin at the end of PCI and before the onset of oral antiplatelet effect may create a vulnerable window for stent thrombosis. Future trials to evaluate definitive role of bivalirudin in STEMI patients should look for if early stent thrombosis could be mitigated by extending infusion of bivalirudin after PCI or using potent antiplatelet agents, including intravenous drugs [89]. Non-STEMI is a different clinical setting, with less thrombin generation and with a lag of 48–72 h between the administration of antiplatelet treatment and coronary intervention. In this latest clinical scenario, the role of bivalirudin appears safe and effective.

## 9. Conclusions

Thrombin plays an essential role in the genesis of acute coronary syndrome because it is involved in both platelet and coagulation system activation. Bivalirudin is an effective anticoagulation treatment with predictable linear dose response to avoid hemorrhagic events. In non-STEMI patients, clinical trials and guidelines agree on the efficacy and safety of bivalirudin over treatment with heparin in PCI. However, the evidence for STEMI patients shows more controversy presenting higher rates of early stent thrombosis, that does not compensate the reduction of bleeding events with bivalirudin. Multicenter randomized clinical trials with radial access and potent P_2_Y_12_ drugs are needed to clarify the role of bivalirudin in STEMI patients. Until then, clinicians should evaluate thrombotic and hemorrhagic risk of ACS patients to achieve the optimal antithrombotic therapy.

## Figures and Tables

**Table 1:** Randomised clinical trials of bivalirudin in patients with STEMI.

Main Trials	HORIZONS-AMI [68]		EUROMAX [69]		HEAT-PPCI [71]		BRIGHT [72]			BRAVE 4 [73]	
**Year**	2008		2013		2014		2015			2014	
**Enrolment**	2005–2007		2010–2013		2012–2013		2012–2013			2009-2013	
**Centres**	123		65		1		82			3	
**Treatment**	Bivalirudin: 0.75 mg/kg bolus followed by an infusion of 1.75 mg/kg/h for the duration of PCI. Heparin: UFH 60 UI/kg bolus with additional bolus to target ACT 200–250 s.		Bivalirudin: 0.75 mg/kg bolus followed by an infusion of 1.75 mg/kg/h for at least 4 h after (0.25–1.75 mg/kg/h). Heparin: UFH 100 UI/kg bolus without GPI or 60 UI/kg with GPI; or enoxaparin 0.5 mg/kg bolus.		Bivalirudin: 0.75 mg/kg bolus followed by an infusion of 1.75 mg/kg/h for the duration of PCI. Heparin: UFH 70 UI/kg bolus with additional doses if ACT < 200 s.		Bivalirudin: 0.75 mg/kg bolus followed by an infusion of 1.75 mg/kg/h for at least 4 h. After reduced infusion 0.2 mg/kg/h for up 20 h. Heparin: UFH 60 UI/kg bolus with additional doses if ACT < 200 s. Heparin + GPI: UFH 60 UI/kg bolus + tirofiban 10 µg/kg bolus followed by infusion 0.15 µg/kg/min for 18 to 36 h.			Bivalirudin + Prasugrel: Prasugrel loading dose of 60 mg + 10 mg daily. Bivalirudin: 0.75 mg/kg bolus followed by an infusion of 1.75 mg/kg/h for the duration of PCI. Heparin + Clopidogrel: Clopidogrel loading dose 600 mg + 75 mg daily. UFH 70–100 UI/kg	
	**HORIZONS-AMI [68]**		**EUROMAX [69]**		**HEAT-PPCI [71]**		**BRIGHT [72]**			**BRAVE 4 [73]**	
	**Bivalirudin**	**Heparin**	**Bivalirudin**	**Heparin**	**Bivalirudin**	**Heparin**	**Bivalirudin**	**Heparin**	**Heparin + GPI**	**Bivalirudin**	**Heparin**
**Patients**	1800	1802	1089	1109	905	907	735	729	730	271	277
**Age**	59.8 (26.0–92.3)	60.7 (21.6–91.6)	61 (52–71)	62 (52–72)	62.9 (53.7–74.0)	63.6 (54.0–73.8)	57.3 ± 11.6	58.1 ± 11.7	58.2 ± 11.8	61.4 (51.9–71.7)	61.4 (52.9–71.5)
**Male**	77.1%	76.1%	74.7%	77.6%	71.0%	73.0%	82.7%	81.6%	82.1%	76%	79%
**Clopidogrel**	95.7%	95.1%	50.0%	51.5%	11.8%	10.0%	100%	100%	99.9%	3.7%	90.2%
**Prasugrel**	0%	0%	30.8%	28.9%	27.3%	27.6%	0%	0%	0%	94.6%	7.1%
**Ticagrelor**	0%	0%	19.2%	19.4%	61.2%	62.7%	0%	0%	0%	0%	0%
**GPI use**	7.2%	94.5%	11.5%	69.1%	13.5%	15.5%	4.4%	5.6%	100%	3.0%	6.1%
**Radial access**	5.7%	5.4%	47.7%	46.3%	80.3%	82.0%	78.4%	79.0%	78.2%	<1%	<1%
**Drug-eluting stent**	NA	NA	57.1%	55.9%	79.7%	79.8%	99.3%	99.3%	99.6%	82.3%	82.3%
**PCI perform**	93.2%	92.2%	87.1%	85.4%	83.0%	81.6%	98.4%	98.6%	98.9%	88.6%	86.6%
**NACE**	9.2%	12.1%	5.1%	8.5%	8.7%	5.7%	8.8%	13.2%	17.0%	15.6%	14.5%
**Bleeding events**	4.9%	8.3%	2.6%	6.0%	3.5%	3.1%	4.1%	7.5%	12.3%	14.1%	12.0%

UHF. Unfractionated Heparin. PCI: Percutaneous coronary intervention. ACT: Activated clotting time. GPI: Glycoprotein IIb/IIIa inhibitors. NACE: Net Adverse Clinical Events at 30-days.

**Table 2:** Randomised clinical trials of bivalirudin in patients with NON-STEMI.

Main Trials	PROTECT-30 [74]		ACUITY [75]			ISAR-REACT [76]	
**Year**	2006		2007			2011	
**Enrolment**	2003–2004		2003–2007			2006–2011	
**Centres**	100		450			8	
**Treatment**	Bivalirudin: 0.75 mg/kg bolus followed by an infusion of 1.75 mg/kg/h for the duration of PCI and 4 h after at physician’s discretion. Heparin: UFH 50 UI/kg bolus with additional bolus to target ACT 200–250 s + epifibatide. LMWH: Bolus of 0.5 mg/Kg + epifibatide.		Bivalirudin alone: 0.75 mg/kg bolus followed by an infusion of 1.75 mg/kg/h. Bivalirudin + GPI: 0.75 mg/kg bolus followed by an infusion of 1.75 mg/kg/h GPI: Epifibatide or Tirofiban. Heparin + GPI: UFH 60 UI/kg bolus plus infusion of 12 UI/kg/h to target ACT 200–250 s or enoxaparin 1 mg/kg twice daily plus bolus 0.3-0.75 mg/kg i.v. during PIC. GPI: epifibatide or tirofiban.			Bivalirudin: 0.75 mg/kg bolus followed by an infusion of 1.75 mg/kg/h for the duration of PCI. Heparin: UFH 70 UI/kg bolus with additional doses if ACT < 200 s + GPI (abciximab 0.25 mg/kg bolus + 0.125 µg/kg/min for 12 h).	
	**Bivalirudin**	**Heparin**	**Bivalirudin**	**Bivalirudin + GPI**	**Heparin + GPI**	**Bivalirudin**	**Heparin**
**Patients**	284	573	2619	2609	2561	860	862
**Age**	59.7 ± 9.8	60.0 ± 11.1	63 (30–92)	62 (21–95)	63 (25–91)	67.5 ± 11.2	67.5 ± 10.8
**Male**	68.3%	66.0%	73.0%	74.0%	73.0%	76.8%	76.9%
**Clopidogrel**	NA		100%	100%	100%	100%	100%
**Prasugrel**	NA		0%	0%	0%	0%	0%
**Ticagrelor**	NA		0%	0%	0%	0%	0%
**GPI use**	3.0%	99%	9.0%	97.0%	97.0%	0%	100%
**Radial access**	NA		6%			<1% (6 patients)	
**Drug-eluting stent**	79%		60.0%	60.0%	61.0%	88.2%	88.9%
**PCI performed**	100%	100%	56.8%	56.7%	56%	99.8%	99.8%
**NACE**	* 1.43%	1.33%	12.0%	15.0%	13.0%	11.0%	10.9%
**Bleeding events**	0.4%	2.5%	4.0%	8.0%	7.0%	2.6%	4.6%

UHF. Unfractionated Heparin. PCI: Percutaneous coronary intervention. ACT: Activated clotting time. GPI: Glycoprotein IIb/IIIa inhibitors. NACE: Net Adverse Clinical Events. * Primary outcome: coronary flow reserve.

**Table 3:** Recommendations of clinical guidelines for STEMI and non-STEMI patients.

**ACS**	**2012 ESC GUIDELINES [77]**	**2013 AHA/ACC GUIDELINES [78]**	**2014 REVASCULARIZATION GUIDELINES [80]**	**NICE Guidelines July 2013 [79]**
**Stemi Patients**	Bivalirudin with use of GP IIb/IIIa blocker restricted to bailout) is recommended over UFH and a GP IIb/IIIa blocker.	Bivalirudin with or without prior treatment with UFH is recommended.	Bivalirudin 0.75 mg/kg i.v. bolus followed by i.v. infusion of 1.75 mg/kg/h for up 4 h after the procedure.	Bivalirudin in combination with aspirin and clopidogrel is recommended for the treatment of adults with STEMI undergoing primary PCI.
	**Class I, Level B**	**Class I, Level B**	**Class IIa, Level A**	
**ACS**	**2015 ESC GUIDELINES [82]**	**2014 AHA/ACC GUIDELINES [83]**	**2014 REVASCULARIZATION GUIDELINES [80]**	**NICE Guidelines March 2010 [81]**
**Non-Stemi Patients**	Bivalirudin (0.75 mg/kg bolus, followed by 1.75 mg/kg/h for up to 4 h after the procedure) is recommended as alternative to UFH plus GP IIb/IIIa receptor inhibitor during PCI.	Bivalirudin 0.10 mg/kg loading dose followed by 0.25 mg/kg per hour (only in patients managed with an early invasive strategy) continued until diagnostic angiography or PCI, with only provisional use of GP IIb/IIIa inhibitor, provided the patient is also treated with DAPT.	Bivalirudin (0.75 mg/kg bolus, followed by 1.75 mg/kg/h for up to 4 h after the procedure) is recommended as alternative to UFH plus GP IIb/IIIa receptor inhibitor during PCI.	As an alternative to the combination of UFH plus GPI, consider bivalirudin for patients who: Are at intermediate or higher risk of adverse cardiovascular events (predicted 6 moth mortality above 3%) Are not already receiving GPI or fondaparinux Are scheduled to undergo angiography within 24 h of admission.
	**Class I, Level A**	**Class I, Level B**	**Class I, Level A**	

ACS: Acute coronary syndrome; UHF: Unfractionated heparin; GP: Glycoprotein; PCI: Percutaneous coronary intervention; DAPT: Dual antiplatelet therapy.

## References

[1] Capodanno D., de Caterina R. (2015). Bivalirudin for acute coronary syndromes: Premises, promises and doubts. Thromb. Haemost..

[2] Alexopoulos D., Stavrou K., Koniari I., Gkizas V., Perperis A., Kontoprias K., Vogiatzi C., Bampouri T., Xanthopoulou I. (2014). Ticagrelor *vs*. prasugrel one-month maintenance therapy: Impact on platelet reactivity and bleeding events. Thromb. Haemost..

[3] Gao F., Shen H., Wang Z.J., Yang S.W., Liu X.L., Zhou Y.J. (2015). Risk and benefit of direct oral anticoagulants or PAR-1 antagonists in addition to antiplatelet therapy in patients with acute coronary syndrome. Thromb. Res..

[4] Fuster V., Badimon J., Chesebro J.H., Fallon J.T. (1996). Plaque rupture, thrombosis, and therapeutic implications. Haemostasis.

[5] He L.W., Dai W.C., Li N.G. (2015). Development of Orally Active Thrombin Inhibitors for the Treatment of Thrombotic Disorder Diseases. Molecules.

[6] Srivastava S.L., Goswami L.N., Dikshit D.K. (2005). Progress in the design of low molecular weight thrombin inhibitors. Med. Res. Rev..

[7] Mann K.G., Brummel K., Butenas S. (2003). What is all that thrombin for?. J. Thromb. Haemost..

[8] Ansell J. (2007). Factor Xa or thrombin: Is factor Xa a better target?. J. Thromb. Haemost..

[9] Huber K., Bates E.R., Valgimigli M., Wallentin L., Kristensen S.D., Anderson J.L., Lopez Sendon J.L., Tubaro M., Granger C.B., Bode C. (2014). Antiplatelet and anticoagulation agents in acute coronary syndromes: What is the current status and what does the future hold?. Am. Heart J..

[10] Cavender M.A., Sabatine M.S. (2014). Bivalirudin *versus* heparin in patients planned for percutaneous coronary intervention: a meta-analysis of randomised controlled trials. Lancet.

[11] Straub A.L., Roehrig S., Hillisch A. (2011). Oral, direct thrombin and factor Xa inhibitors: the replacement for warfarin, leeches, and pig intestines?. Angew. Chem. Int. Ed. Engl..

[12] Ferrante G., Valgimigli M., Pagnotta P., Presbitero P. (2015). Bivalirudin *versus* heparin in patients with acute myocardial infarction: A meta-analysis of randomized trials. Catheter. Cardiovasc. Interv..

[13] Showkathali R., Natarajan A. (2012). Antiplatelet and antithrombin strategies in acute coronary syndrome: State-of-the-art review. Curr. Cardiol. Rev..

[14] Mackman N. (2008). Triggers, targets and treatments for thrombosis. Nature.

[15] Fuster V., Badimon L., Badimon J.J., Chesebro J.H. (1992). The pathogenesis of coronary artery disease and the acute coronary syndromes. Part I. N. Engl. J. Med..

[16] Epstein F., Fuster V., Badimon L., Badimon J.J., Chesebro J.H. (1992). The pathogenesis of coronary artery disease and the acute coronary syndromes. Part II. N. Engl. J. Med..

[17] Falk E., Shah P.K., Fuster V. (1995). Coronary plaque disruption. Circulation.

[18] Furie B., Furie B.C. (1992). Molecular and cellular biology of blood coagulation. N. Engl. J. Med..

[19] Goel M.S., Diamond S.L. (2004). Factor VIIa-mediated tenase function on activated platelets under flow. J. Thromb. Haemost..

[20] De Cristofaro R., de Candia E. (2003). Thrombin domains: structure, function and interaction with platelet receptors. J. Thromb. Thrombolysis.

[21] Coughlin S.R., Camerer E., Hamilton J.R., Colman R.W., Mardeer V.J., Clowes A.W., George J.N., Goldhaber S.Z. (2006). Protease-activated receptors in hemostasis, thrombosis, and vascular biology. Protease Activated Receptors in Hemostasis, Thrombosis, and Vascular Biology, Hemostasis and Thrombosis: Basic Principles and Clinical Practice.

[22] Angiolillo D.J., Capodanno D., Goto S. (2010). Platelet thrombin receptor antagonism and atherothrombosis. Eur. Heart J..

[23] Coughlin S.R. (2000). Thrombin signalling and protease-activated receptors. Nature.

[24] Monroe D.M., Hoffman M., Roberts H.R. (2002). Platelets and thrombin generation. Arterioscler. Thromb. Vasc. Biol..

[25] Weitz J.I., Hudoba M., Massel D., Maraganore J., Hirsh J. (1990). Clot-bound thrombin is protected from inhibition by heparin-antithrombin III but is susceptible to inactivation by antithrombin III-independent inhibitors. J. Clin. Unvest..

[26] Kumar R., Béguin S., Hemker H.C. (1994). The influence of fibrinogen and fibrin on thrombin generation evidence for feedback activation o f the clotting system by clot bound thrombin. Thromb. Haemost..

[27] Davie E.W., Kulman J.D. (2006). An overview of the structure and function of thrombin. Semin. Thromb. Hemost..

[28] Weitz J.I. (2014). Insights into the role of thrombin in the pathogenesis of recurrent ischaemia after acute coronary syndrome. Thromb. Haemost..

[29] Bajzar L., Manuel R., Nesheim M.E. (1995). Purification and characterization of TAFI, a thrombin-activable fibrinolysis inhibitor. J. Biol. Chem..

[30] Sakharov D.V., Plow E.F., Rijken D.C. (1997). In the mechanism of the antifibrinolytic activity of plasma carboxypeptidase B. J. Biol. Chem..

[31] Ewald G.A., Eisenberg P.R. (1995). Plasmin-mediated activation of contact system in response to pharmacological thrombolysis. Circulation.

[32] Eisenberg P.K., Miletich J.R., Sobel B.E., Jaffe A.S. (1998). Differential effects of activation of prothrombin by streptokinase compared with urokinase and tissue-type plasminogen activation (t-PA). Thromb. Res..

[33] Seitz R., Pelzer H., Immel A., Egbring R. (1993). Prothrombin activation by thrombolytic agents. Fibrinolysis.

[34] Glover C.J., McIntire L.V., Leverett L.B., Hellums J.D., Brown C.H., Natelson E.A. (1974). Effect of shear stress on clot structure formation. Trans. Am. Soc. Artif. Intern. Organs.

[35] Fenton F.W., Ofosu F.A., Brezniak D.V., Hassouna H.I. (1998). Thrombin and antithrombotics. Semin. Thormb. Hemost..

[36] Tricoci P., Huang Z., Held C., Moliterno D.J., Armstrong P.W., van de Werf F., White H.D., Aylward P.E., Wallentin L., Chen E. (2012). TRACER Investigators. Thrombin-receptor antagonist vorapaxar in acute coronary syndromes. N. Engl. J. Med..

[37] Burke D.A., Warraich H.J., Pinto D.S. (2012). Which antithrombin for whom? Identifying the patient population that benefits most from novel antithrombin agents. Curr. Cardiol. Rep..

[38] De Caterina R., Husted S., Wallentin L., Andreotti F., Arnesen H., Bachmann F., Baigent C., Huber K., Jespersen J., Kristensen S.D. (2013). Parenteral anticoagulants in heart disease: Current status and perspectives (Section II). Position paper of the ESC Working Group on Thrombosis-Task Force on Anticoagulants in Heart Disease. Thromb. Haemost..

[39] Rao S.V., Ohman E.M. (2010). Anticoagulant therapy for percutaneous coronary intervention. Circ. Cardiovasc. Interv..

[40] Lee L., Chew D. (2012). Promise of factor Xa inhibition in acute coronary syndromes. Curr. Cardiol. Rep..

[41] Gibson C.M., Murphy S.A., Montalescot G., Morrow D.A., Ardissino D., Cohen M., Gulba D.C., Kracoff O.H., Lewis B.S., Roguin N. (2007). Percutaneous coronary intervention in patients receiving enoxaparin or unfractionated heparin after fibrinolytic therapy for ST-segment elevation myocardial infarction in the ExTRACT-TIMI 25 trial. J. Am. Coll. Cardiol..

[42] Montalescot G., White H.D., Gallo R., Cohen M., Steg P.G., Aylward P.E., Bode C., Chiariello M., King S.B., Harrington R.A. (2006). Enoxaparin *versus* unfractionated heparin in elective percutaneous coronary intervention. N. Engl. J. Med..

[43] Yusuf S., Mehta S.R., Chrolavicius S., Afzal R., Pogue J., Granger C.B., Budaj A., Peters R.J., Bassand J.P., Wallentin L., Joyner C., Fox K.A. (2006). Comparison of fondaparinux and enoxaparin in acute coronary syndromes. N. Engl. J. Med..

[44] Yusuf S., Mehta S.R., Chrolavicius S., Afzal R., Pogue J., Granger C.B., Budaj A., Peters R.J., Bassand J.P., Wallentin L. (2006). Effects of fondaparinux on mortality and reinfarction in patients with acute ST-segment elevation myocardial infarction: The OASIS-6 randomized trial. JAMA.

[45] Bates S.M., Weitz J. (1998). Direct thrombin inhibitors for treatment of arterial thrombosis: potential differences between bivalirudin and hirudin. Am. J. Cardiol..

[46] Ansell J.E. (2016). Universal, class-specific and drug-specific reversal agents for the new oral anticoagulants. J. Thromb. Thrombolysis.

[47] Gómez-Outes A., Suárez-Gea M.L., Lecumberri R., Terleira-Fernández A.I., Vargas-Castrillón E. (2015). Direct-acting oral anticoagulants: pharmacology, indications, management, and future perspectives. Eur. J. Haematol..

[48] Heidbuchel H., Verhamme P., Alings M., Antz M., Hacke W., Oldgren J., Sinnaeve P., Camm A.J., Kirchhof P. (2013). European Heart Rhythm Association Practical Guide on the use of new oral anticoagulants in patients with non-valvular atrial fibrillation. Europace.

[49] Eikelboom J.W., Weitz J.I. (2015). “Realworld” use of non-vitamin K antagonist oral anticoagulants (NOACs): Lessons from the Dresden NOAC Registry. Thromb. Haemost..

[50] Markwardt F. (1991). Past, present, and future of hirudin. Haemostasis.

[51] Maragonore J.M., Bourdon P., Jablonsky J., Ramachandran K.L., Fenton J.W. (1990). Design and characterization of hirulogs: A novel class of bivalent peptide inhibitors of thrombin. Biochemistry.

[52] De Caterina R., Husted S., Wallentin L., Andreotti F., Arnesen H., Bachmann F., Baigent C., Huber K., Jespersen J., Kristensen S.D. (2013). General mechanisms of coagulation and targets of anticoagulants (Section I). Position Paper of the ESC Working Group on Thrombosis-Task Force on Anticoagulants in Heart Disease. Thromb. Haemost..

[53] Witting J.L., Bourdon P., Brenziak D.V., Fenton J.W. (1992). Thrombin-specific inhibition by an slow cleavage of hirulog-1. Biochem. J..

[54] Parry M.A., Maraganore J.M., Stone S.R. (1994). Kinetic mechanism for the interaction of Hirulog with thrombin. Biochemistry.

[55] Bates S.M., Weitz J.I. (2000). The mechanism of action of thrombin inhibitors. J. Invasive Cardiol..

[56] Warkentin T.E., Greinacher A., Koster A. (2008). Bivalirudin. Thromb Haemost..

[57] Gladwell T.D. (2002). Bivalirudin: a direct thrombin inhibitor. Clin. Ther..

[58] (2005). Angiomax™ (Package insert) Parsippany.

[59] Robson R., White H., Aylward P., Frampton C. (2002). Bivalirudin pharmacokinetics and pharmacodynamics: Effect of renal function, dose, and gender. Clin. Pharmacol. Ther..

[60] Lidón R.M., Théroux P., Juneau M., Adelman B., Maraganore J. (1993). Initial experience with a direct antithrombin, Hirulog, in ustable angina. Anticoagulant, antithrombotic and clinical effects. Circulation.

[61] Topol E.J., Bonan R., Jewitt D., Sigwar U., Kakkar V.V., Rothman M., de Bono D., Ferguson J., Willerson J.T., Strony J. (1993). Use of a direct antithrombin, Hirulog, in place of heparin during coronary angioplasty. Circulation.

[62] Lui H.K. (2000). Dosage, pharmacological effects and clinical outcomes for bivalirudin in percutaneous coronary intervention. J. Invasive Cardiol..

[63] Bittl J.A., Strony J., Brinker J.A., Ahmed W.H., Meckel C.R., Chaitman B.R., Maraganore J., Deutsch E. (1995). Adelman B, for the Hirulog Angioplasty Study Investigatiors. Treatment with bivalirudin (Hirulog) as compared with heparin during coronary angioplasty for unstable or postinfarction angina. N. Engl. K. Med..

[64] Pötzch B., Hund S., Madlener K., Seelig C., Riess C.F., Greinacher A., Müller-Berghaus G. (1997). Monitoring of r-hirudin anticoagulation during cardiopulmonary bypass: Assessment of the whole blood ecarin clotting time. Thromb. Haemost..

[65] Koster A., Chew D., Gründel M., Bauer M., Kuppe H., Spiess B.D. (2003). Bivalirudin monitored with the ecarin clotting time for anticoagulation during cardiopulmonary bypass. Anesth. Analg..

[66] Zhang D., Wang Z., Zhao X., Lu W., Gu J., Cui Y. (2011). Pharmacokinetics, pharmacodynamics, tolerability and safety of single doses of bivalirudin in healthy chinese subjects. Biol. Pharm. Bull..

[67] Fox I., Dawson A., Loynds P., Eisner J., Findlen K., Levin E., Hanson D., Mant T., Wagner J., Maraganore J. (1993). Anticoagulant activity of Hirulog, a direct thrombin inhibitor, in humans. Thromb. Haemost..

[68] Stone G.W., Witzenbichler B., Guagliumi G., Peruga J.Z., Brodie B.R., Dudek D., Kornowski R., Hartmann F., Gersh B.J., Pocock S.J. (2008). Bivalirudin during primary PCI in acute myocardial infarction. N. Engl. J. Med..

[69] Steg P.G., van’t Hof A., Hamm C.W., Clemmensen P., Lapostolle F., Coste P., Ten Berg J., van Grunsven P., Jan Eggink G., Nibbe L. (2013). Bivalirudin started during emergency transport for primary PCI. N. Engl. J. Med..

[70] Clemmensen P., Wiberg S., van’t Hof A., Deliargyris E.N., Coste P., Ten Berg J., Cavallini C., Hamon M., Dudek D., Zeymer U. (2015). Acute stent thrombosis after primary percutaneous coronary intervention: insights from the EUROMAX trial (European Ambulance Acute Coronary Syndrome Angiography). JACC Cardiovasc. Interv..

[71] Shahzad A., Kemp I., Mars C., Wilson K., Roome C., Cooper R., Andron M., Appleby C., Fisher M., Khand A. (2014). Unfractionated heparin *versus* bivalirudin in primary percutaneous coronary intervention (HEAT-PPCI): An open-label, single centre, randomised controlled trial. Lancet.

[72] Han Y., Guo J., Zheng Y., Zang H., Su X., Wang Y., Chen S., Jiang T., Yang P., Chen J. (2015). Bivalirudin *vs.* heparin with or without tirofiban during primary percutaneous coronary intervention in acute myocardial infarction: The BRIGHT randomized clinical trial. JAMA.

[73] Schulz S., Richardt G., Laugwitz K.-L., Morath T., Neudecker J., Hoppmann P., Mehran R., Gershlick A., Tolg R., Anette Fiedler K. (2014). Prasugrel plus bivalirudin *vs.* clopidogrel plus heparin in patients with ST-segment elevation myocardial infarction. Eur. Heart J..

[74] Gibson C.M., Morrow D.A., Murphy S.A., Palabrica T.M., Jennings L.K., Stone P.H., Lui H., Bulle T., Lakkis N., Kovach R. (2006). A randomized trial to evaluate the relative protection against post-percutaneous coronary intervention microvascular dysfunction, ischemia, and inflammation among antiplatelet and antithrombotic agents: The PROTECT-TIMI-30 trial. J. Am. Coll. Cardiol..

[75] White H.D., Chew D.P., Hoekstra J.W., Miller C.D., Pollack C.V., Feit F., Lincoff A., Bertrand M., Pocock S., Ware J. (2008). Safety and efficacy of switching from either unfractionated heparin or enoxaparin to bivalirudin in patients with non-ST-segment elevation acute coronary syndromes managed with an invasive strategy: Results from the ACUITY (Acute Catheterization and Urgent Intervention Triage strategY) trial. J. Am. Coll. Cardiol..

[76] Sibbing D., Bernlochner I., Schulz S., Massberg S., Schömig A., Mehilli J., Kastrati A. (2012). Prognostic value of a high on-clopidogrel treatment platelet reactivity in bivalirudin *versus* abciximab treated non-ST-segment elevation myocardial infarction patients. ISAR-REACT 4 (Intracoronary Stenting and Antithrombotic Regimen: Rapid Early Action for Coronary Treatment-4) platelet substudy. J. Am. Coll. Cardiol..

[77] Steg P.G., James S.K., Atar D., Badano L.P., Blömstrom-Lundqvist C., Borger M., Di Mario C., Dickstein K., Ducrocq G., Fernandez-Aviles F. (2012). ESC Guidelines for the management of acute myocardial infarction in patients presenting with ST-segment elevation. Eur. Heart J..

[78] O’Gara P.T., Kushner F.G., Ascheim D.D., Casey D.E., Chung M.K., de Lemos J.A., Ettinger S., Fang J., Fesmire F., Franklin B. (2013). 2013 ACCF/AHA guideline for the management of ST-elevation myocardial infarction: Executive summary: A report of the American College of Cardiology Foundation/American Heart Association Task Force on Practice Guidelines: Developed in collaboration with the American College of Emergency Physicians and Society for Cardiovascular Angiography and Interventions. Catheter. Cardiovasc. Interv..

[79] National Clinical Guideline Centre (UK) (2013). Myocardial Infarction with ST-Segment Elevation: The Acute Management of Myocardial Infarction with ST-Segment Elevation [Internet].

[80] Windecker S., Kolh P., Alfonso F., Collet J.-P., Cremer J., Falk V., Filippatos G., Hamm C., Head S., Juni P. (2014). 2014 ESC/EACTS Guidelines on myocardial revascularization: The Task Force on Myocardial Revascularization of the European Society of Cardiology (ESC) and the European Association for Cardio-Thoracic Surgery (EACTS)Developed with the special contribution of the European Association of Percutaneous Cardiovascular Interventions (EAPCI). Eur. Heart J..

[81] National Clinical Guideline Centre (UK) (2010). Unstable Angina and NSTEMI: The Early Management of Unstable Angina and Non-ST-Segment-Elevation Myocardial Infarction.

[82] Roffi M., Patrono C., Collet J-P., Mueller C., Valgimigli M., Andreotti F., Bax J.J., Borger M.A., Brotons C., Chew D.P. (2016). 2015 ESC Guidelines for the management of acute coronary syndromes in patients presenting without persistent ST-segment elevation: Task Force for the Management of Acute Coronary Syndromes in Patients Presenting without Persistent ST-Segment Elevation of the European Society of Cardiology (ESC). Eur. Heart J..

[83] Amsterdam E.A., Wenger N.K., Brindis R.G., Casey D.E., Ganiats T.G., Holmes D.R., Jaffe A., Jneid H., Kelly R., Kontos M., Levine G. (2014). 2014 AHA/ACC Guideline for the Management of Patients with Non-ST-Elevation Acute Coronary Syndromes: a report of the American College of Cardiology/American Heart Association Task Force on Practice Guidelines. J. Am. Coll Cardiol..

[84] Valgimigli M., Frigoli E., Leonardi S., Rothenbühler M., Gagnor A., Calabrò P., Garducci S., Rubartelli P., Briguori C., Andò G. (2015). Bivalirudin or Unfractionated Heparin in Acute Coronary Syndromes. N. Engl. J. Med..

[85] Farag M., Gorog D.A., Prasad A., Srinivasan M. (2015). Bivalirudin *versus* unfractionated heparin: A meta-analysis of patients receiving percutaneous coronary intervention for acute coronary syndromes. Open Heart.

[86] Bittl J.A., He Y., Lang C.D., Dangas G.D. (2015). Factors Affecting Bleeding and Stent Thrombosis in Clinical Trials Comparing Bivalirudin With Heparin During Percutaneous Coronary Intervention. Circ. Cardiovasc Interv..

[87] Navarese E.P., Schulze V., Andreotti F., Kowalewski M., Kołodziejczak M., Kandzari D.E., Rassaf T., Gorny B., Brockmeyer M., Meyer C. (2015). Comprehensive meta-analysis of safety and efficacy of bivalirudin *versus* heparin with or without routine glycoprotein IIb/IIIa inhibitors in patients with acute coronary syndrome. JACC Cardiovasc. Interv..

[88] Piccolo R., de Biase C., D’Anna C., Trimarco B., Piscione F., Galasso G. (2015). Early stent thrombosis with bivalirudin in patients undergoing percutaneous coronary intervention. A meta-analysis of randomised clinical trials. Thromb. Haemost..

[89] Alexander W. (2015). Bivalirudin Versus Heparin: A Fight Far From Finished?: Efficacy, Safety, and Cost Remain Battlegrounds for the Treatment Of ST-Segment Elevation Myocardial Infarction. Pharm. Ther..

